# Patients’ experiences of the BetterBack model of care for low back pain in primary care – a qualitative interview study

**DOI:** 10.1080/17482631.2020.1861719

**Published:** 2021-01-03

**Authors:** Paul Enthoven, Fredrik Eddeborn, Allan Abbott, Karin Schröder, Maria Fors, Birgitta Öberg

**Affiliations:** a Department of Health, Medicine and Caring Sciences, Division of Prevention, Rehabilitation and Community Medicine, Unit of Physiotherapy, Linköping University, Linköping, Sweden; b Rehab West, Region Östergötland, and Department of Health, Medicine and Caring Sciences, Linköping University, Linköping, Sweden; c Department of Activity and Health, and Department of Health, Medicine and Caring Sciences, Linköping University, Linköping, Sweden

**Keywords:** Low back pain, physiotherapy, qualitative interview, primary care, health care professionals, education, common-sense model, reassurance, self-management, self-efficacy

## Abstract

**Purpose**: The aim of this study was to describe patient experiences of received primary care for low back pain (LBP) according to the BetterBack Model of Care (MoC) with a focus on illness beliefs and self-management enablement.

**Methods**: Individual interviews were conducted with 15 adults 4–14 months after receiving treatment according to the BetterBack MoC for LBP in primary care in Sweden. Data were analysed using content analysis.

**Results**: When analysing the data, the following theme emerged; “Participant understanding of their treatment for low back pain and self-management strategies—a matter of support systems”, comprising the following categories: “*Knowledge translation*”, “*Interaction and dialogue”, “The health care professional support*” and “*Form organization*”. Participants experienced that they had better knowledge about their LBP and received tools to better manage their health condition. The participants expressed good communication with the treating physiotherapist and provided suggestions to further improve the treatment of LBP.

**Conclusions**: Participants experienced that they had gained new knowledge about their health problems and after the treatment they had the tools to handle their back problems. This suggests that the BetterBack MoC may be used as a basis for a support system to provide valuable tools for self-management for patients with low back pain.

## Introduction

Low back pain (LBP) is a large public health problem and the major cause of disability worldwide (GBD Disease and Injury Incidence and Prevalence Collaborators, 2017). In Sweden and many other countries, most patients with LBP are treated in primary care. Many patients improve within a short period but a significant proportion report recurrences or continuous pain even after several years (Enthoven et al., 2004; Oberg et al., 2003).

According to the Swedish board of health and welfare, good health care should be patient-centred, knowledge-based, safe, efficient, equal, and accessible (National Board of Health and Welfare, 2009). Patient-centred care should focus on self-management and healthy lifestyles as a means of restoring and maintaining function and optimizing participation in society (Buchbinder et al., 2018). Self-management can be defined as “the tasks that individuals must undertake to live with one or more chronic conditions”. These tasks include having the confidence to deal with medical management, role management, and emotional management of their conditions (Adams et al., 2004).

Evidence-based practice suggests that patients with musculoskeletal pain problems in primary care can be managed effectively with treatments such as self-management advice, exercise therapy, and psychosocial interventions (Babatunde et al., 2017; National Clinical Guideline Centre (NICE), 2016; Sundhedsstyrelsen, 2016). Recently, international guidelines for LBP have been locally adapted to the Swedish primary care setting providing a foundation for the development of the BetterBack Model of Care (MoC) (Abbott et al., 2018). Implementation of the BetterBack MoC aims to change the behaviour of the treating health care professionals (HCPs) towards applying a more biopsychosocial management coherent with best practice guidelines (Foster et al., 2018) and to provide patients with tools to better self-manage episodes of LBP (Abbott et al., 2018; Leventhal et al., 2016). It is hypothesized that this may lead to improved patient-reported LBP related illness beliefs, pain, physical function and quality of life.

The common-sense model of self-regulation (CSM) suggests that HCPs can support patients to self-manage their illness in a number of ways: provide information about the condition; provide access to review; advice on how to monitor the course of the condition; and through the provision of a personal condition-specific action plan, which recommends when and how to adjust treatment and/or seek timely help during deterioration (Leventhal et al., 2016).

The BetterBack MoC includes tools supporting patient assessment as well as the design and progression of individualized physiotherapy. Core patient educational interventions include individualized information at initial and follow-up visits, a standardized brochure about back pain and self-management, and group education aiming to rectify maladaptive LBP beliefs, reassure good prognosis and maintenance of physical activity. Other core interventions include home-based and/or group-based exercise interventions used to further support the maintenance of physical activity and enablement of self-management behaviours.

The implementation of the BetterBack MoC was planned a priori to be evaluated both at health care professional level and patient level (Briggs et al., 2016). A recent evaluation of the physiotherapists´ (PTs’) confidence, attitudes and beliefs in managing patients with LBP before and after a multifaceted implementation of the BetterBack MoC showed that PTs´ confidence and biopsychosocial orientation increased after implementation and may have the potential to improve management of LBP in primary care (Schroder et al., 2020). In addition to health care provider related measures, patients’ experiences of health services can also be regarded as a key component in evaluating the quality of care (De Silva, 2013). Interviewing patients might provide additional knowledge on how the MoC is perceived and may identify areas/components with potential for improvement. Therefore, the aim of the current study was to describe patient experiences of receiving care according to the BetterBack MoC for LBP in primary care with a focus on illness beliefs and self-management enablement.

## Methods

The study is part of a larger project investigating the effectiveness and implementation of the BetterBack MoC for LBP in primary care in Sweden (Abbott et al., 2018). In the larger project, 500 patients with LBP were recruited from all 15 primary care physiotherapy clinics in the Östergötland public health care region and 278 of these patients were randomized to treatment according to the BetterBack MoC (Abbott et al., 2018). The design of the present study is a qualitative interview study on the experiences of a subset of patients after receiving treatment for LBP according to the BetterBack MoC. The Regional Ethical Review Board of Linköping has approved the study (Dnr 2017/35-31, Dnr 2018/202-32) (Abbott et al., 2018). All participants signed an informed consent form after receiving oral and written information. The trial was registered at ClinicalTrials.gov, NCT03147300, 2017. The study is reported in line with the COnsolidated criteria for REporting Qualitative research (COREQ) checklist for qualitative studies (Tong et al., 2007).

### Participants and setting

All participants had previously been treated according to the BetterBack MoC for a first-time or recurrent episode of benign LBP with or without radiculopathy in the Östergötland public health care region in south-east Sweden (Abbott et al., 2018). Further Key inclusion criteria were men and women 18–65 years and fluent in Swedish. Key exclusion criteria were current diagnosis of malignancy, spinal fracture, infection, cauda equina syndrome, ankylosing spondylitis or systemic rheumatic disease, previous malignancy during the past 5 years; current pregnancy or previous pregnancy up to 3 months before consideration of inclusion; patients who fulfil the criteria for multimodal/multiprofessional rehabilitation for complex long-standing pain, severe psychiatric diagnosis and spinal surgery the last 2 years. Since the BetterBack MoC includes a variety of interventions offered to patients depending on the therapist’s assessment findings and patient needs, the current study was based on a purposive sample to provide variability in the treatment they received: 1) Core interventions (individualized information about assessment and treatment plan at initial and follow-up visits, a standardized brochure about LBP and self-management, as well as a home exercise programme, 2) Core interventions and group education, 3) Core interventions and group exercise, and 4) Core interventions and group exercise and group education, where 1) corresponded to the least comprehensive and 4) to the most comprehensive treatment according to the BetterBack MoC. We expected this to reflect participants with different severities of LBP (length of and risk for persistent disabling LBP (Hill et al., 2008)). Furthermore, the goal was to include both women and men of various ages.

Potential participants were identified from the BetterBack MoC study database and recruited from two primary care clinics; the smaller clinic had nine PTs and the larger clinic had 16 PTs working according to the BetterBack MoC. The primary care clinics sent out information about the study by postal mail to 30 eligible subjects. In total 26 subjects were contacted by telephone by FE and 15 (58%) agreed to participate and were booked for an interview.

### Data collection

An interview guide with open and semi-structured questions was developed by the research group and used to remind the interviewer of topics to include (Appendix A). The guide covered questions about the participants’ communication with HCPs, their ability to manage their back pain, and their experiences with different parts of the BetterBack MoC. Each interview began with the open-ended question “What are your experiences of the treatment for your back pain?” To reach a deeper understanding, follow-up questions were asked, such as “Could you tell me more?”

The first and second authors, none of whom had been involved in the participants’ treatment and both fluently in Swedish, conducted the interviews, which occurred between May and September 2018, 4–14 (median 8.5) months after the participants finished treatment for LBP. One interviewer asked the questions and the other sat beside and could ask complementary questions to ensure no relevant information was missed. All interviews were conducted at the participants’ primary care centre, except for one interview that was conducted at Linköping University. The interviews lasted 19–41 (median 32) minutes. The interviews were audiotaped and transcribed verbatim by an independent transcription expert. After each interview, the interviewers had a brief talk about their impression of the interview, which was also audio-recorded as a memory support and was later used by the interviewers during the analysis phase. The first interview was seen as a pilot interview to test the interview guide (Patton, 2015), but since only small grammatical changes were made to the interview guide, this interview was included in the analysis.

### Data analysis

The interview data were analysed using qualitative content analysis (Graneheim & Lundman, 2004), which is a systematic method useful when analysing data on people’s experiences and reflections (Downe-Wamboldt, 1992). The analysis started with reading the interviews in their entirety to gain an overview of the content and to identify meaning-bearing units corresponding to the aim of the study. Then, codes (core sentences and words) were extracted from meaning-bearing units (abbreviated part of the text). Each meaning unit was labelled with a code. The coding process was done with the software OpenCode 4.03available from https://www.umu.se/en/department-of-epidemiology-and-global-health/research/open-code2/.

All authors were physiotherapists and had many years’ experience treating patients with LBP. FE and MF had the experience of working according to the BetterBack MoC. The authors PE and FE coded the entire material and discussed the codes. The authors KS and MF each coded three interviews for triangulation (Patton, 2015). In a meeting PE, KS and MF discussed the codes until consensus was reached. The codes were sorted and grouped into subcategories and categories in discussion among the authors. Based on the categories an overall theme emerged. The subcategories, categories and overall theme were discussed and negotiated among the authors in a final discussion until consensus was reached. Table I gives an example of the coding process. During the analysis, the first author made field notes of reflections and interpretations.

**Table I.** Subcategories and examples of quotes in the category “Knowledge translation”

Categories	Subcategories	Examples of quotes
Knowledge translation	Information and explanations about back pain	I was happy with the explanation
		My back muscles were weak
		The pain is related to too heavy work
	Information and explanations about management of low back pain	It is good to walk every day
		My muscles need to become stronger
		The pain would diminish by the exercises

## Results

The sample included nine women and six men with a median of 47 (min 25—max 62) years of age, large variability in the treatment they received, and different severities of LBP. This was judged to be a large enough sample to provide a variety of experiences and to allow enough depth in the analysis (Malterud, 2016; Patton, 2015). Additional pertinent background data are provided in Table II. Eight participants from one primary care centre and seven participants from another primary care centre were interviewed. All 15 participants had received individualized information about their assessment and treatment plan at initial and follow-up visits, a standardized brochure about LBP and self-management, as well as a home exercise programme as core interventions. Dependent upon the risk of persisting disabling LBP (Hill et al., 2008) and individual preferences, the participants were offered supervised group-based exercise sessions 2 times per week over 6 weeks as well as a group-based pain education session.

**Table II.** Participants’ characteristics

Participant	Sex	Age, years	Content of the BetterBack MoC received	STarT Back risk profile^a^
1	M	60	Core interventions^a^	Low risk
2	W	36	Core interventions^a^ + group ED	Medium risk
3	M	54	Core interventions^a^ + group ED & group EX	Medium risk
4	W	46	Core interventions^a^ + group ED & group EX	Medium risk
5	M	29	Core interventions^a^ + group ED	High risk
6	W	60	Core interventions^a^ + group ED	High risk
7	M	35	Core interventions^a^	Low risk
8	W	59	Core interventions^a^	Low risk
9	W	62	Core interventions^a^ + group EX	Low risk
10	M	28	Core interventions^a^ + group ED	High risk
11	W	57	Core interventions^a^ + group ED & group EX	Medium risk
12	W	56	Core interventions^a^ + group ED & group EX	Low risk
13	M	39	Core interventions^a^ + group ED	Medium risk
14	W	25	Core interventions^a^	Medium risk
15	W	52	Core interventions^a^	Medium risk

M = Man, W = Woman, ED = Education, EX = Exercise.^a^ Core interventions = Individualized information about assessment and treatment plan at initial and follow-up visits, a standardized brochure about LBP and self-management, as well as a home exercise programme.

^a^Risk group qualification according to the STarT Back Tool.

**Figure 1.**
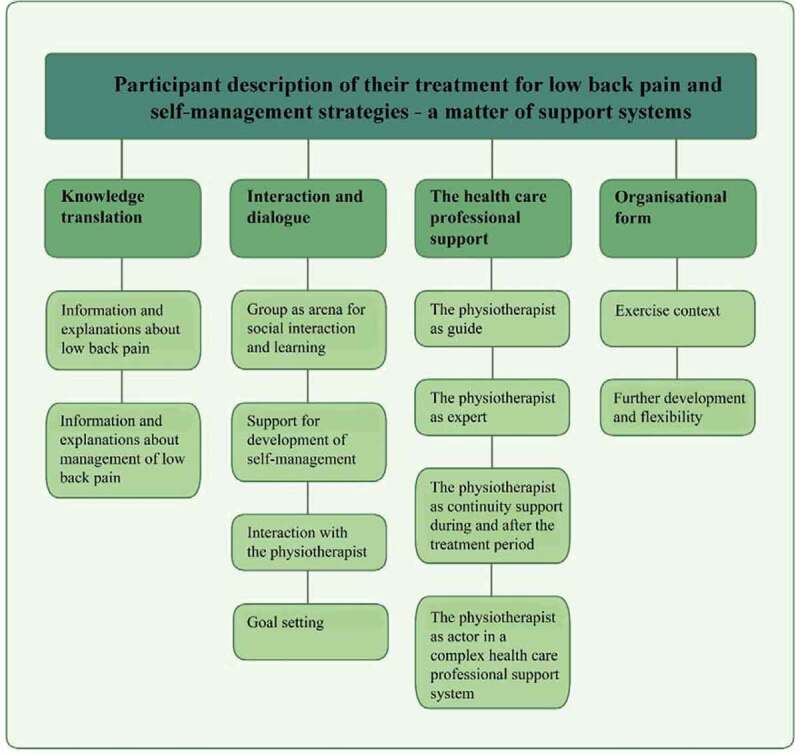
Overall theme, categories, and subcategories of the qualitative content analysis (no text below Figure 1)

An overall theme was conceptualized: Participant description of their treatment for low back pain and self-management strategies—a matter of support systems (see Figure 1). Participants described that they had received support tools to better self-manage their condition. From the analysis four categories with subcategories emerged: 1) Knowledge translation, 2) Interaction and dialogue, 3) The health care professional support and 4) Organizational form. The emergent categories are presented below, with quotations in italics.

### Knowledge translation

#### Information and explanations about low back pain

Participants experienced that they received information about their back pain resulting in more knowledge and a better understanding of their back pain. They thought the information was tailored to their personal complaints. Getting more knowledge created, among other things, calm individuals because they were worried that they had a more serious illness.

“ … well, you get less worried, because you always think that there is something very dangerous in the back, because that is what I thought at first. I thought, “now I have got cancer in the back”, but later I realized that it was not that, … ” (Participant 11)

To facilitate knowledge transfer, PTs had used pedagogical tools such as pictures, and anatomical models, which the participants experienced increased their understanding of their back pain. Information was transmitted directly between the PT and the participant, and via group education, which was appreciated.

“They showed me on boards and stuff what it could be … so I got more information than I had before about it … ” (Participant 11)

“You were told things that you didn’t know before, like muscle memory or pain memory … that I have taken with me a little afterwards … so it was very informative and good … It was probably more the knowledge to know more about why you might have pain or how it works in the body and how it responds, so it has most to do with the whole back.” (Participant 10)

Most participants were pleased with the explanation for their back pain. They found the explanation understandable and acceptable. But, also critical views on the given explanation were expressed with beliefs in other causes of back pain.

“.I was happy with the explanation (the PT) gave me and why it should be good and how to do and so, … ” (Participant 8)

“a part I can agree with is that the abdominal muscles and back muscles were weak and so on, but at the same time, it cannot be because of the abdominal muscles and the back muscles the pain comes, I have very often lumbago and then you wonder if it is related or if it is something else … ” (Participant 2)

#### Information and explanations about management of low back pain

Participants re-evaluated their illness beliefs and felt they had been given a fair explanation of the treatment plan by the PT.

“Took with me that it is not dangerous to have pain, it is just the body’s way of telling that it is a little too much, that in many cases you can prevent and that you also can get relief by exercising or acting the right way and what counts is to learn to live with what you have.” (Participant 12)

Participants revealed that they had increased knowledge on how they could self-manage their back pain and that they had changed their lifestyle. However, some participants wanted more help with how to handle obstacles in everyday life. Other participants expressed that they would not have known what to do with their back if they had not received the treatment.

“Before, I did nothing like exercise or anything, now I do a lot of training and things like that because I feel that is beneficial for my own health, it is my health that is important.” (Participant 5)

“I mean, if there are things one should think about when doing certain things in the home especially, at home and in the garden and so on” (Participant 12)

The participants received a brochure about back pain and found the content to be informative and relevant at the time. It was also a help to inform relatives and others about their back pain and the treatment. Several kept the brochure at home, and sometimes read the brochure again.

“It (the brochure) said a lot and it puts one’s finger on these things that you both did and didn’t know and how things are related. So it was good … Above all that you have the fundamentals, so that you can talk to your surroundings, explain to them how things are interconnected, because there is really nothing you can see on me … after all pain is not visible.” (Participant 12)

### Interaction and dialogue

#### Group as arena for social interaction and learning

Most participants who received group exercise and group education found it to be among the best parts of the treatment programme. Meeting other patients with similar complaints that could understand their problems was highly appreciated. They learned from each other, e.g., by exchanging information on how to deal with everyday activities, which attributed to better self-management of their back pain. Stories from patients with more severe problems gave them other perspectives on their own back pain. They also gathered strength to better manage their own problems. However, not all participants wanted to discuss their back pain with other patients.

“It was good because you noticed that you are not alone, sometimes it feels like you are alone with this trouble, but then there are many others who have the same trouble, there are others who have worse.” (Participant 2)

“I thought the most important with the course was that you saw and heard the others and what they did to get pain relief and that you shared with other participants.” (Participant 6)

“No, we did not talk about our back pain during group exercise, I did that with my PT.” (Participant 9)

#### Support for development of self-management

The participants felt they had been given tools to better self-manage their back pain after the treatment.

“I’m really happy, I really am, it has helped me a lot. Even though I am not quite good, I have the tools to, yes, actually relieve … ” (Participant 11)

The participants described they developed ways to deal with their problems such as doing specific exercises tailored for that participants’ back pain, use aids in everyday life or split up different activities to diminish the load on the body. The participants felt that this help created an independence that they knew how to handle their back problems before they arose or when they arose and decreased worries that the back pain would get worse.

“If I am standing and do a certain activity for a very long time, I try to think and stop and try to stretch a little or, make a little change so I don’t do the same monotonous stuff all the time, because then I know I’m getting problems later. So things like that I have taken with me from here.” (Participant 10)

“. now I can stop it (back pain) … I’m not at all worried that I’ll get it back … if I get pain I know what to do.” (Participant 11)

#### Interaction with the physiotherapist

Participants experienced good contact and interaction with the PT. They felt that the PTs were empathetic, and they could discuss their back pain and the treatment. For example, good interaction was perceived for adaptation of their tailored exercise programme, e.g., when it was time to progress the training or alternatively if the participants’ complaints deteriorated.

“I think the understanding, listening to the patient, being aware of what need I have, that felt very good. The PT I had was very good at giving the right help, so to say, so you find the right way … I was never afraid to ask and the PT explained very well.” (Participant 15)

“Some exercises were maybe a little bit too hard at first and when we did them together, we both noticed that we might have to start one step further down. So, we could still talk about what suited me. That I thought was great.” (Participant 14)

#### Goal setting

Participants thought it was valuable to be part of the goal setting together with the PT.

“. I got to be part of the goal setting. The PT asked what I was thinking and then (the PT) thought it was good … it was probably my goals and then (the PT) developed the exercises after that, so that we would achieve it.” (Participant 14)

There were views that participants and the PT did not talk a lot about goal setting, at least did not discuss specific tasks/goals. Participants could experience that it would have been good to discuss more concrete goals. Other participants expressed that for them more concrete targets/goals were not needed.

“I don’t remember if we were talking about any goal really, … goals are also such big words … sometimes you don’t say “what is your goal?” but we talked about it, the work and being able to work.” (Participant 8)

“No, I’m pretty good at hacking them myself. Being able to lie down and get up can be a partial goal, I don’t have to have anyone else who sets it as a goal, the stated goals I fixed myself.” (Participant 13)

### The health care professional support

#### The physiotherapist as guide

Participants experienced the PT as someone they could trust and that guided them throughout the entire rehabilitation period keeping them safe and positive about future prognosis. The guidance included both supervision and monitoring of the treatment.

“When I started treatment, I was very worried and sad, because I still had back pain and didn’t really know what I could and couldn’t do or if I could work and so on. Then I got a PT who guided me that I could really trust, which I thought was great, so I felt safe with the PT.” (Participant 8)

#### The physiotherapist as expert

The participants saw the PT as the trusted expert with a lot of knowledge about back pain in general, competence to understand their specific back pain problems and to give appropriate advice on how to deal with their problems. Participants were grateful for the help they received.

“The PTs are knowledgeable, they listen, so they do not have a one-way view of how things are, but they listen and take in and they show pictures and explain and … I think they are generally very good to have around you, nice.” (Participant 12)

#### The physiotherapist as a continuity support during and after the treatment period

The participants appreciated continuity in the support from the PT during the performance of the programme. Some participants expressed that it was more helpful for them to have contact with the same (“their own”) PT all the time. Others preferred to meet other PTs who could give new directions.

“ … when you came one day there was someone who was up there and then the next time maybe it was somebody else, then they gave me new tips” … “So, it was even good that it was a little different … ” (Participant 11)

In general, the participants were satisfied with the follow-up and number of treatments during the treatment period necessary for continuity. A critical view expressed was that there was a lack of follow-up and evaluation during the treatment period. Follow-ups could be good for evaluation, goal setting and for defining smaller steps to achieve larger goals later. Some views illustrated that follow-ups were not needed. Participants however appreciated that they always could contact the primary care clinic again if needed.

”Yes, for me, I think it would have been good to have that continuity (more visits). Then I would have, felt that I had a goal, …, got some kind of evaluation that I had gotten better or so, or if I have stand still in development, and maybe got something new to take with me.” (Participant 7).

“We didn’t think it made any sense for me to go here anymore, but I always had the opportunity to call here if it got worse, then I would have called and could have come here immediately.” (Participant 1)

Some participants expressed a desire to continue with exercise at the primary care clinic even after the treatment period. They felt the clinic was a secure place to perform exercises in contrast to fitness studios or gym facilities outside the clinic that had completely different clients. Other reasons for wanting to come back were to get new exercises or to keep up the motivation to continue with exercising.

“What I wished for was that I could come back sometime, … maybe every third month, come back on a return visit and got some new exercises, so that you had kept it going. After the last visit I did exercises at home … after a while you do not continue with exercises when there is no feedback.” (Participant 7)

#### The physiotherapist as actor in a complex health care professional support system

Many participants only had contact with a physiotherapist, but some also described that they had visits to for example, chiropractors, occupational health care and for example, a GP to prescribe sick leave and/or pain medication. Caregivers could give different advices which could be confusing and frustrating.

“Seeking a chiropractor was my own choice, there was nothing that stood in the way, the doctor thought, but then I would wait a few weeks before I contacted you (physiotherapy), because that was the first question I had “Will there be any contact with the PT or so?” “Yes, yes, later on, we’ll see,” (the GP) said. (Participant 13)

“.but (the PT) said I would turn to a doctor and I was sent here and there from there.” (Participant 2)

The interaction with different HCPs could make participants unsure about who was responsible for what and perceived as an obstacle for how to decide upon diagnosis and treatment.

“.the rehab bit seems to be a bit free from medical care, the regular GP care, is there any dialogue between PTs and doctors? Or it is just a referral you get? “(Participant 12)

### Organizational form

#### Exercise context

Participants experienced a great advantage in doing their exercise programme at home because it suited their other activities. Others found it a disadvantage to do the exercise programme at home, because they felt that they did not have the capability to perform the program without support from the primary care clinic. A disadvantage with doing the exercises at home could be a diminishing motivation to continue doing the exercises.

“I don’t need equipment I rather do the programme at home and at work, anywhere … It works great.” (Participant 4)

“Instead of coming here (the clinic) and doing the exercises, I did them at home. I felt that … it fitted better in everyday life to be able to do it at home … It worked pretty well the first half of the year … then you lost it and the motivation disappeared … that is the disadvantage (of home exercise), if I had trained here, the PTs could have said if I might try something else, so it could have been an advantage to be here instead.” (Participant 10)

#### Further development and flexibility

Participants were interested in getting even more information about their back pain and treatment. Some participants found the group education too basic and therefore not adjusted to their personal needs. They suggested to have different sessions with different levels of information so patients that already knew a lot about LBP could learn more. Suggestions were made to discuss other subjects, such as diet (with the goal of weight loss), mental and social conditions, and pregnancy linked to LBP and exchange of experiences with other patients.

“Yes, yes, because it also affects, because I mean stress itself is, after all, an onset that causes us to get more pain if we already have pain somewhere.” (Participant 12)

“You might open up more for discussion and be able to give each other tips “this is how I think, this exercise I think works great for me” … now it was more teaching” (Participant 13)

To be able to continue with their self-management, participants found it valuable to have direct access to the information, either written or in other forms.

“ … if I am in pain, I can go through the papers at home so I know what to do all the time. It is good also that I have saved all the papers at home. Then you see, then I can go through the exercises once more at home.” (Participant 5)

“Yes, maybe video, to see how I should perform my exercises” (Participant 15)

Some participants wanted increased access to the primary care clinic to be better able to participate in different parts of the treatment. Another reason was that participants wanted to have the possibility for return visits to encourage and support their self-management ability

“.I could not be there (in the clinic) at all exercise times because I have this job.” (Participant 4)

## Discussion

Participants generally experienced that they had gained new knowledge about LBP, and had acquired tools to handle their back problems, which is in line with the desired effects of the BetterBack MoC. The analysis revealed four categories regarding the participants' experiences of their treatment for LBP: *Knowledge translation* played an important role in providing participants with information and explanation about their back pain and its management. *Interaction and dialogue* dealt with communication and integration of knowledge and action. *The health care professional support* described the different roles of the PT and other HCPs as support for the participant’s treatment plan and development of self-management. *The organizational form* described possible influences on how the process is perceived with regards to delivering the content of the BetterBack MoC, as well as its accessibility and continuity. An important part was good communication with the treating physiotherapist and that the participants felt they were listened to. Diverse views emerged on components of the BetterBack MoC and their organization. This indicated the importance of providing flexibility in the support systems to optimize patient-centred primary care for LBP.

The participants in the present study expressed that the PT provided explanations about LBP and its management and provided a continuity in the information throughout the whole treatment period. This information about LBP was important for the participants to feel reassured that they did not have a serious disease. A meta-analysis suggests that there is moderate- to high-quality evidence that patient education in primary care can provide long-term reassurance and reduce the number of health care visits for patients with acute or subacute LBP (Traeger et al., 2015). Illness concerns may lead patients to avoid normal activities that cause discomfort (Bishop et al., 2015; Crombez et al., 2012; Glattacker et al., 2013b; Wertli et al., 2014). Therefore, the earlier HCPs help patients identify misconceptions and develop coherent illness presentations about their LBP, the greater the chance that the patient may recover and avoid chronic disorders (Wertli et al., 2014).

Some participants in the current study expressed that they had doubts about the explanation of LBP they received from the PT despite relief of concerns about possible sinister causes. Participants could express that it was hard to really understand their back pain, despite now having the tools to control their back pain. Previous research including a meta-synthesis of 38 qualitative studies highlighted that patients may experience a generic LBP diagnosis as insufficient to understand their symptoms. Furthermore, this may add to further distress, loss of capacity, disempowerment and lower adherence to treatment. Instead, patients expect empathy, listening, respect and individualized explanations of potential causal factors to be able to better understand and manage their symptoms (Dima et al., 2013; Macneela et al., 2015).

Back pain is however a complex biopsychosocial condition often not related to specific identifiable spinal abnormalities which can make it difficult to explain the exact cause to the patient (Airaksinen et al., 2006; Buchbinder et al., 2018). Instead of focusing on spinal abnormalities, the BetterBack MoC utilizes a balance model metaphor explaining that LBP is a result of physical, emotional and/or social demands temporarily exceeding patients’ physical, emotional, and/or cognitive capacity. Education about LBP and its management together with exercise interventions can help tip the scale back again so that the patient's capacity exceeds demands (Abbott et al., 2018). According to the CSM, if the patient gains an adequate understanding of LBP and its management, this may increase his/her motivation to implement the information and exercises provided by the physiotherapist (Leventhal et al., 2016). The BetterBack MoC education material uses a biopsychosocial perspective because this approach provides greater knowledge about the condition, physical activity level and satisfaction with patients at long-term follow-up compared to lecture material with a biomedical content (Meng et al., 2017). Also, a meta-analysis on neck and back pain found that education with a more biopsychosocial perspective had positive effects while education only including information on biomechanics and posture did not (Ainpradub et al., 2016).

Participants in the current study with moderate-high risk of prolonged LBP activity limitations who received group education expressed that it was very rewarding. This because patients with similar problems could better understand them, they could exchange experiences and advice, and it gave them a new perspective on their own situation. Some participants however found the group education too basic and therefore not adjusted to their personal needs and suggested having different sessions with different levels of information. In comparison with previous research, Pietilä-Holmner et al. (Pietila Holmner et al., 2018) found that sharing experiences in group sessions of patients with chronic pain had led to fellowship and less feelings of loneliness for certain participants. However, participation in group sessions could also be experienced as negative, because of fear that their pain might be reinforced if they listened to other people’s pain stories. King et al. (King et al., 2018) found that patients with chronic LBP showed varying levels of reconceptualization (≈ changes in beliefs about back pain) 3 weeks after receiving one session of group education. The degree of reconceptualization was influenced by the patients’ previous beliefs and the estimated relevance of the information, and was related to the perceived benefit, and patients without perceived benefit did not change their perceptions. King et al. (King et al., 2018) suggest that having more sessions and combining the education with other interventions may improve the benefits. Translated into CSM terminology, group education may heighten the possibility for patients to reflect upon and influence illness and treatment beliefs, and content of personalized condition-specific action plans.

According to a review study group exercise and individual exercise have similar effects on pain and disability (O’keeffe et al., 2017). Due to high appreciation by the participants in the current study and potentially lower health care costs, group exercise should be offered to all patients with moderate-high risk of prolonged LBP activity limitations. However, patient preferences should also be taken into consideration (King et al., 2018; O’keeffe et al., 2017; Slade et al., 2009) because some participants wanted to do exercises at home and some could not attend the group sessions, e.g., due to working hours. As exercise may work via multiple mechanisms, future studies should also include analysis to better understand which patients respond best to individual or group exercise. Further, education combined with exercise has a larger effect on pain than education alone (Louw et al., 2016). In chronic pain, education combined with other therapies is likely to improve both self-management and self-efficacy (Joypaul et al., 2019).

Goal setting is an important element in modern rehabilitation and should include exploring the patients’ global meaning (i.e., fundamental beliefs, goals and attitudes) after which a meaningful overall rehabilitation goal can be formulated (Dekker et al., 2020). After that, the patient and HCP can set specific rehabilitation goals to achieve the overall rehabilitation goal. The participants' stories in the current study gave the impression that goal setting was not the programme component that received the most attention. Many participants did not consider this a problem because for them the obvious goal was to become pain-free. Others missed the goal setting, especially discussing specific concrete goals. Stenner et al. (2016) found little attention to goal setting in patients receiving exercise therapy for LBP and suggested this could be related to a practitioner-centred care instead of patient-centred care. To improve patient’s involvement in their treatment and motivation to follow treatment advice, it may be good that the patients together with the physiotherapist formulate some timely, concrete goals. According to the CSM, if the outcome of behaviour/action plan is consistently appraised as being in the direction of the goal, the behaviour makes sense. Repeated over time this may lead to higher adherence to the behaviour and reduce fear-avoidance behaviour (Bunzli et al., 2017). Goal setting does not necessarily lead to improved results (Glattacker et al., 2013a), but a recent study using combined education and patient-led goal setting found positive effects on long-term back pain and disability (Gardner et al., 2018).

Most participants felt that the treatment helped to facilitate everyday activities and contributed to increased physical activity, but some wanted more help with managing everyday life. Participants experienced that after the treatment they had the tools to better manage their life independently, which is one desired effect of the BetterBack MoC. Literature suggests that avoiding activities may increase the risk of more pain and ill health (Crombez et al., 2012).

Considering that a previous period of LBP is an important risk factor for a new period with pain (Taylor et al., 2014) and about one-third or more of patients seeking care report a recurrence within 1 year (Enthoven et al., 2004; Da Silva et al., 2017), having these support tools may help to reduce the number and effects of future episodes of back pain for many patients. An increased ability of the patient to handle his/her LBP may also lead to less need for health care and sick leave and thereby reduce financial stress on society (Olafsson et al., 2018). According to CSM action plans for self-management need to be linked to a specific condition (Leventhal et al., 2016) and for an optimal result the content should be described in detail and communicated between the patient and the HCP (Mansell et al., 2016).

Most participants in the current study felt it was easy to communicate with the PT, showed trust in the PT, and found the PTs knowledgeable. These are all factors that may strengthen the interaction between the patient and the PT, and thereby shared decision-making (Andersen et al., 2019). In previous interview studies, patients with non-specific LBP described that they value that PTs are motivational, supportive and explain the causes of their discomfort and treatment (Slade et al., 2009). Bernhardsson et al. (2017) found that trust and confidence in the PTs skills and competence stimulated patients with a musculoskeletal disorder to actively engage in their treatment, including wanting to participate in decision-making. In the current study some participants' conceptions about cause and treatment could be based on insufficient information, e.g., HCPs provided different information. This could confuse participants which was also found in another study (Andersen et al., 2019). Considering that participants also wanted to have information more readily available, the exercises and other important information could be made available to patients using online information, which is also promoted by other researchers/clinicians (Slater et al., 2020; World Health Organisation, 2019).

Some participants in the current study described that they needed more time to process the information given. In an interview study patients on a waiting list for spinal surgery expressed that the period of pre-surgery conservative treatment gave them valuable time for reassurance and to reflect on treatments (Lindback et al., 2019). In the current study several participants felt that the treatment time was not enough for the training they performed to become a habit. Also, participants wanted to know if adjustment to their home exercises was needed due to changed conditions, and more follow-up was also seen as an opportunity to adjust goal setting. A study on non-specific LBP showed that patients found it important to be able to perform their exercises correctly (Slade et al., 2009). Furthermore, a study on asthma found that later follow-up offered an opportunity to review behaviours and potentially help patients break ineffective self-management “habits” and promote new strategies (Daines et al., 2020). In terms of the CSM, follow-up visits may be a possibility to improve the patients’ representations of their illness and treatment, and adjust the individualized action plan. However, not limiting the number of visits may also lead to making the patient dependent on the caregiver, which must be taken into consideration in the shared decision-making process.

Some participants expressed that they did not want to question the opinion of the PT, because the PT was the expert “that surely knows better than me”. In an interview study patients with lumbar disk herniation expressed it was difficult to have another opinion than the clinician regarding the decision about surgery (Andersen et al., 2019). This power imbalance between patient and HCP may influence the decision-process negatively. Some participants in the current study wanted to be more involved in decision-making than others, which was also found in frail elderly patients within goal-setting meetings in rehabilitation (Rose et al., 2019), and highlights that also individual factors may have an influence on the shared decision-making process (Andersen et al., 2019).

### Methodological considerations

The research group consisted of both women and men of different ages, with both short and long experience of treating patients with LBP and both clinically and academically active authors. This diversity permitted different perspectives on the findings and is a strength of the study. Involvement of other professions than physiotherapists could have strengthened the work.

All interviews in the study were conducted by two persons. The one who did not hold the interview had the opportunity to ask follow-up questions in case something needed to be clarified or deepened, which is a strength. Another strength was that the interviewers had the opportunity to discuss their views on the interview afterwards and could discuss the coding since both were familiar with the material, and thereby increasing credibility (Patton, 2015). A disadvantage might be that the interviewee (participant) felt put in an inferior position which has been reported to be a risk with three or more interviewers and if the interview contains more sensitive topics (Patton, 2015). The participants reported no problem or feelings of being put in an inferior position. One interviewer and one other author had experience of working with the BetterBack MoC, ensuring a good understanding of participants’ issues (Tong et al., 2007).

The semi-structured interview guide was based on overarching questions about how participants perceived their treatment for LBP, as well as direct questions about the newly introduced BetterBack MoC. An inductive approach was used where construction of the theme, (sub)categories and codes was driven by the content of the data.

The participants were chosen through purposeful sampling to enhance rich variation in data and to gain credibility (Graneheim & Lundman, 2004). A study strength was the broad sample of participants of different age, sex, length and severity of LBP demanding treatment with different components of the BetterBack MoC, which strengthen the credibility and transferability of the results in primary care in Sweden or internationally. There might be organizational as well as cultural differences between primary care centres that make the result of the current study more or less generalizable. Interviews with participants from more primary care centres would have been preferable, but this was not possible for logistic reasons. The size of the primary care clinic may influence the availability/access to the group education and group exercise of the BetterBack MoC, which may have an effect on the transferability to smaller settings. The same study performed in a society with a different health care system or other norms about LBP might yield different findings.

Although offered different locations almost all participants choose to have the interview at the clinic. In a health care environment participants might be more reserved and feel disempowered talking (Tong et al., 2007), but the participants said it did not bother them, and no doubtful or negative feelings were expressed.

A priori we decided to interview 15 individuals. As doing “enough” interviews depends on several factors such as the study aim, sample specificity, theory, quality of discussion, and analysis strategy (Malterud, 2016), it is hard to know what is correct. Interview 15 showed some new codes, but after interview 13 no new subcategories or categories were identified.

With retrospective interview studies such as this, there is a risk that participants have forgotten how they experienced parts of the treatment, and that their feelings might have changed afterwards. This is hard to influence, but important to take into consideration doing the analysis (Patton, 2015). However, it was judged more important to catch the participants' experiences at a later stage also to study more long-term consequences of the intervention, such as a possible change of behaviour.

When doing interview studies, there is a risk that only interested and positive individuals participate. A strength in our study is that both positive and negative opinions about the treatment were presented.

### Practice implication and future research

Most participants experienced that they had gained new knowledge about their health condition which can improve illness beliefs and treatment outcomes. The BetterBack MoC can be seen as a support system that contains both treatment intervention and interaction between different stakeholders to provide continuity and support for self-management. Additional experimental clinical studies are needed to evaluate if the model of care can improve the patients’ health, care consumption and future clinical course.

## Conclusions

Participants experienced that they had gained new knowledge about their health problems and after the treatment they had the tools to handle their back problems. This suggests that the BetterBack MoC, including knowledge transfer, interaction and dialogue, health care provider support and flexible organization may be used as a basis for a support system to provide valuable tools for self-management for patients with low back pain.

## Appendix A. Interview guide

*Initial question*

1. What are your experiences with the treatment for your back pain?*Other questions.*

2. What did you and your physiotherapist talk about regarding your back pain?—What did you think about the explanation you got for your back pain?—Have you changed your view of your back pain, if so, in what way?3. Did you get an explanation about the treatment you received?—What did you think about the explanation?—How did you experience your treatment (content, arrangement, benefit, etc.)?4. How do you feel you can handle your back pain on your own?—Do you feel that you received help with this during the treatment period? If so, in what way?—Do you have a strategy if the pain comes back? Has the treatment contributed to (a change in) your strategy? If yes, can you tell us more (WHAT in the treatment)?5. Can you be physically active today?—How are your everyday activities, for example, regarding gardening, walking, etc.?—Have you changed your way of being physically active and if so, what has contributed to that (treatment by physiotherapist, pain reduction, something else)? (too little or excess activity?)6. Did you receive a brochure about back pain? If so, what did you think of it?—Do you use it (the brochure)? (in everyday life?)7. Did you participate in the group education on back pain? If so, what did you think of it?—What was good/less good?—What did you take with you (learn) from the group education?8. Did you participate in group exercise? If so, what did you think of it?—The arrangement? Number of participants?—Exercises and change of exercises during the treatment period? The support of the physiotherapist?—Training hours, other? 9. Did you have the possibility to influence your treatment? (exercises, etc.)—Did your therapist listen to you if you wanted to change something?—Have you wanted to influence more/less? 10. Did you set goals for the treatment? Did you do that together with the therapist? (if needed give examples of goals so the participant understands what you are aiming at, e.g., being able to work, walking a certain distance, get less pain …)—Do you think the goals were appropriate/reasonable? 11. How did you experience the contact/communication by the physiotherapist?—Satisfied/not satisfied?—why/why not? (group/individual treatment)?—How did you experience the contact with your therapist(s)? In what way?—How often did you have contact with your therapist? Was that too often/too rare? 12. Is there anything else you would like to add regarding the treatment of your back pain?—Could anything have been done differently?

**Extra material**

**Consolidated criteria for reporting qualitative studies (COREQ): 32-item checklist**

Developed from:

Tong A, Sainsbury P, Craig J. Consolidated criteria for reporting qualitative research (COREQ): a 32-item checklist for interviews and focus groups. *International Journal for Quality in Health Care*. 2007. Volume 19, Number 6: pp. 349–357

**Table:**

No. Item	Guide questions/description	Reported on Page #
**Domain 1: Research team and reﬂexivity**		
*Personal Characteristics*		
1. Interviewer/facilitator	Which author/s conducted the interview or focus group?	Page 7
2. Credentials	What were the researcher’s credentials? E.g. PhD, MD	Page 1
3. Occupation	What was their occupation at the time of the study?	Page 1, 25, 26
4. Gender	Was the researcher male or female?	Page 25
5. Experience and training	What experience or training did the researcher have?	Page 25
*Relationship with participants*		
6. Relationship established	Was a relationship established prior to study commencement?	Page 7
7. Participant knowledge of the interviewer	What did the participants know about the researcher? e.g., personal goals, reasons for doing the research	Page 5, 6
8. Interviewer characteristics	What characteristics were reported about the inter viewer/facilitator? e.g., Bias, assumptions, reasons and interests in the research topic	Page 7, 8, 25, 26
**Domain 2: study design**		
*Theoretical framework*		
9. Methodological orientation and Theory	What methodological orientation was stated to underpin the study? e.g., grounded theory, discourse analysis, ethnography, phenomenology, content analysis	Page 2, 8, 26
*Participant selection*		
10. Sampling	How were participants selected? e.g., purposive, convenience, consecutive, snowball	Page 6
11. Method of approach	How were participants approached? e.g., face-to-face, telephone, mail, email	Page 6
12. Sample size	How many participants were in the study?	Page 6
13. Non-participation	How many people refused to participate or dropped out? Reasons?	Page 6
*Setting*		
14. Setting of data collection	Where was the data collected? e.g., home, clinic, workplace	Page 7
15. Presence of non-participants	Was anyone else present besides the participants and researchers?	No.
16. Description of sample	What are the important characteristics of the sample? e.g., demographic data, date	Page 6, 7, Table II
*Data collection*		
17. Interview guide	Were questions, prompts, guides provided by the authors? Was it pilot tested?	Page 7, 8 Appendix A
18. Repeat interviews	Were repeat interviews carried out? If yes, how many?	No.
19. Audio/visual recording	Did the research use audio or visual recording to collect the data?	Page 7, 8
20. Field notes	Were ﬁeld notes made during and/or after the interview or focus group?	Page 7, 8
21. Duration	What was the duration of the interviews or focus group?	Page 7
22. Data saturation	Was data saturation discussed?	Page 6, 27
23. Transcripts returned	Were transcripts returned to participants for comment and/or correction?	No. Page 27
**Domain 3: analysis and ﬁndings**		
*Data analysis*		
24. Number of data coders	How many data coders coded the data?	Page 9
25. Description of the coding tree	Did authors provide a description of the coding tree?	Page 8 Table I
26. Derivation of themes	Were themes identiﬁed in advance or derived from the data?	Page 9, 26
27. Software	What software, if applicable, was used to manage the data?	Page 8
28. Participant checking	Did participants provide feedback on the ﬁndings?	No Page 27
*Reporting*		
29. Quotations presented	Were participant quotations presented to illustrate the themes/ﬁndings? Was each quotation identiﬁed? e.g. participant number	Page 9–18
30. Data and ﬁndings consistent	Was there consistency between the data presented and the ﬁndings?	Page 9–18
31. Clarity of major themes	Were major themes clearly presented in the ﬁndings?	Page 9–18
32. Clarity of minor themes	Is there a description of diverse cases or discussion of minor themes?	Page 9–18
